# Thoracoscopic right anterior basal segmentectomy with unique anatomic variation: a case report and literature review

**DOI:** 10.3389/fonc.2025.1562303

**Published:** 2025-06-26

**Authors:** Tiancheng Liu, Sishi Huang, Jianbin Zhang, Lili Jin, Qibin Shen

**Affiliations:** ^1^ Department of Thoracic Surgery, Huzhou Central Hospital, Fifth School of Clinical Medicine of Zhejiang Chinese Medical University, Huzhou, Zhejiang, China; ^2^ Department of Thoracic Surgery, Huzhou Central Hospital, Affiliated Central Hospital of Huzhou University, Huzhou, Zhejiang, China; ^3^ Department of Central Laboratory, Huzhou Central Hospital, Fifth School of Clinical Medicine of Zhejiang Chinese Medical University, Huzhou, Zhejiang, China; ^4^ Department of Central Laboratory, Huzhou Central Hospital, Affiliated Central Hospital of Huzhou University, Huzhou, Zhejiang, China

**Keywords:** three-dimensional computed tomography bronchography and angiography, anatomical variation, basal segment, anatomic segmentectomy, lung cancer

## Abstract

**Background:**

Anatomic basal segmentectomy is often regarded as a complex procedure, particularly in the presence of complex anatomical variations.

**Case report:**

Herein, we present a case of a 55-year-old female patient diagnosed with invasive lung adenocarcinoma. Three-dimensional computed tomography bronchography and angiography (3D-CTBA) displayed a unique combined variation in the basal segment of right lower lobe (RLL): The medial basal subsegmental bronchi (BX^7^a+BX^7^t) originated from both the anterior and posterior segmental bronchi; The medial basal subsegmental arteries emanated from the anterior and posterior basal segmental arteries; The medial anterior segmental vein shared a trunk with both the anterior basal and lateral basal subsegmental veins. With precise preoperative planning, thoracoscopic anterior basal segmentectomy was successfully executed.

**Conclusion:**

This case again highlights the importance of 3D-reconstruction in pulmonary segmentectomy. Detailed understanding of anatomic features of segmental bronchi and vessels is imperative for those with complex anatomical variations.

## Introduction

Thoracoscopic basal segmentectomy presents formidable technical challenges for thoracic surgeons due to frequent anatomical variations (1). Preoperative understanding of segmental anatomy is crucial for surgeons to perform precise operations (2). Although numerous variations of the basal segment have been reported by Zhang M et al. (3), the type of variant in this case has rarely been previously reported. With meticulous preoperative planning and accurate intraoperative judgment, thoracoscopic right anterior basal segment (RS^8^) segmentectomy was successfully performed.

## Case report

**Figure 1:**
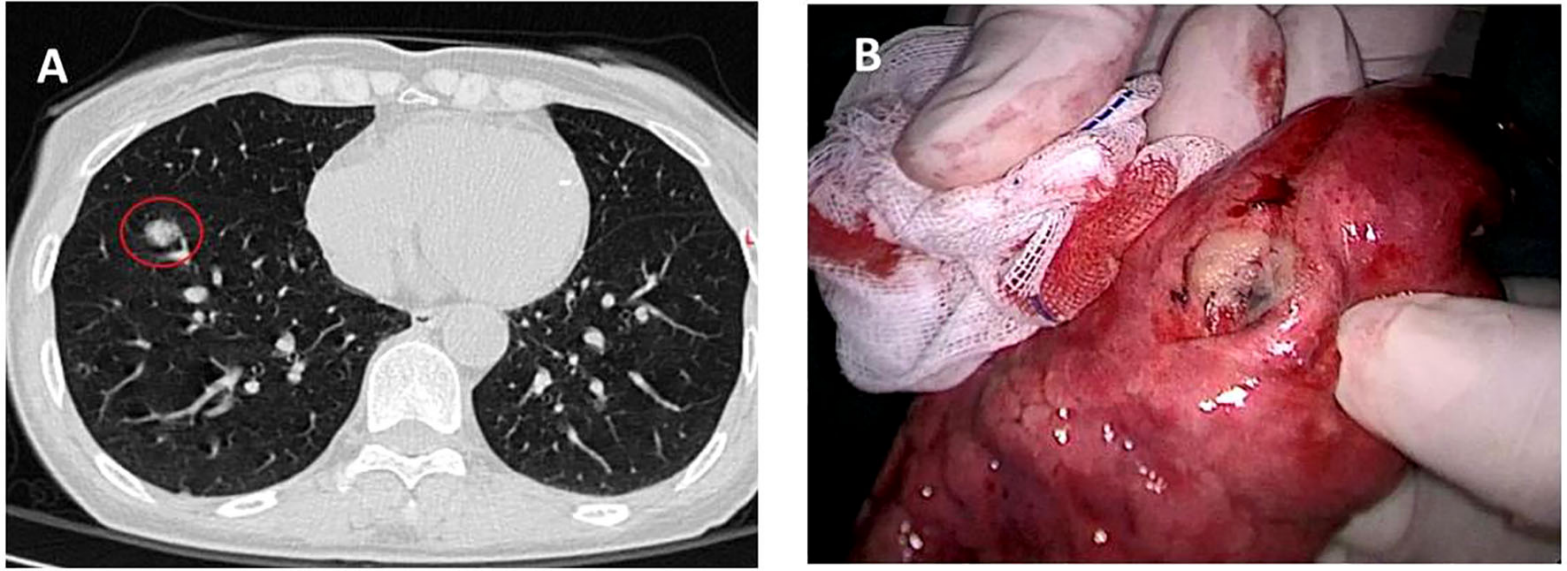
Preoperative chest CT scan and the intraoperative specimen. **(A)** A 13 mm-diameter subsolid nodule in the anterior basal segment of right lower lobe. **(B)** The tumor was 2.5 cm away from the incisal margin.

**Figure 2:**
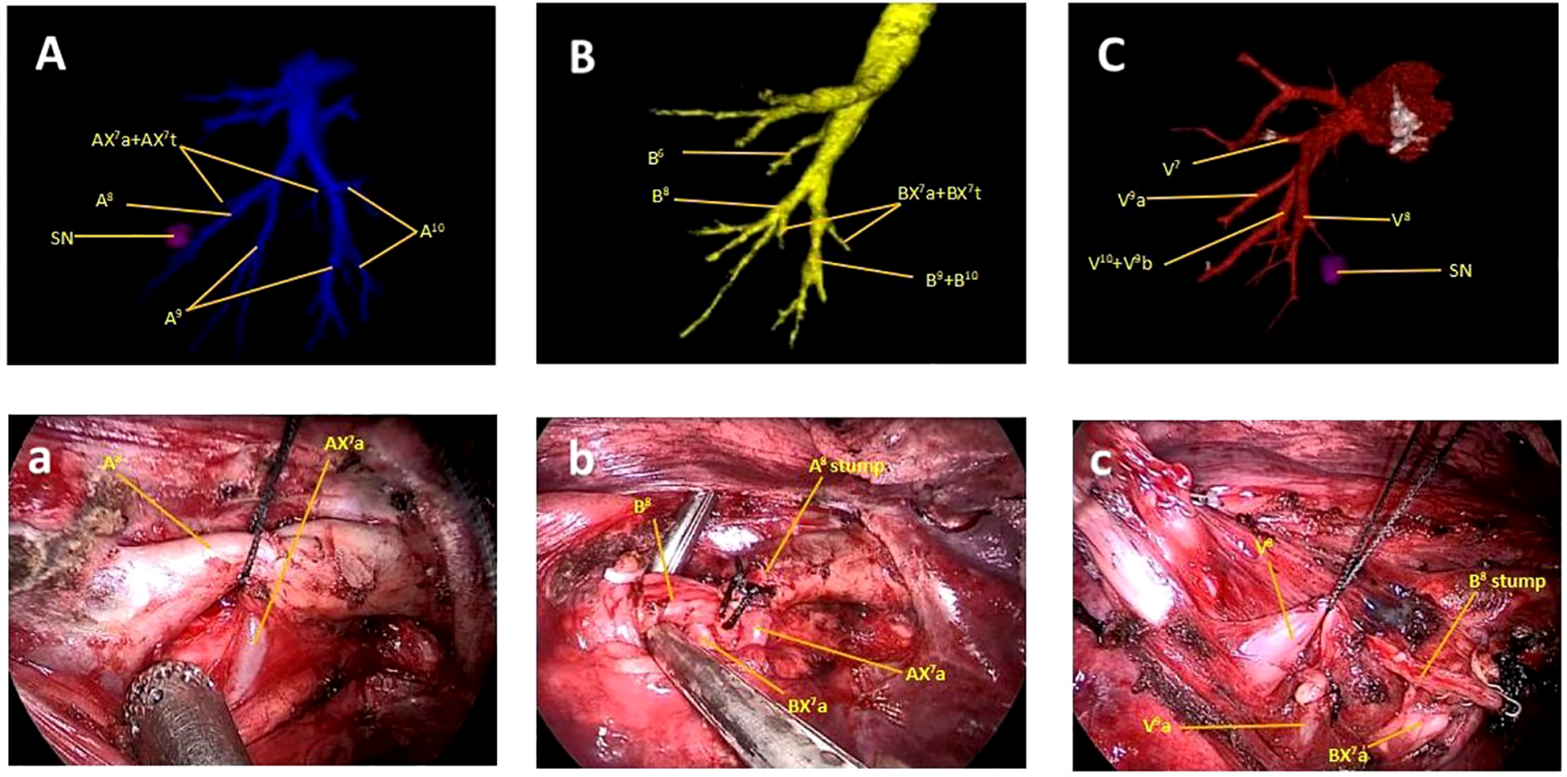
Preoperative 3D-CTBA of the right lower lobe and intraoperative findings. **(A, a)** The AX^7^a emanated from the A^8^. **(B, b)** BX^7^a originated from the B. **(C, c)** The V^7^ shared a trunk with the V^8^ and the V^9^a.

A 55-year-old woman was admitted to our department with a subsolid nodule in the right lower lobe (RLL), which had been under surveillance for five years following its initial detection. The high-resolution computed tomography (HRCT) revealed a 13 mm nodule in the RS^8^ (**Figure 1A**). The patient presented no history of malignant tumors, no disease symptoms, and no family history of lung cancer. No distant metastases were detected. The pulmonary function was normal. A unique variation in the right basal segment was identified via three-dimensional computed tomography bronchography and angiography (3D-CTBA). CT scan was performed using Toshiba Aquillion One 320-row Dual-Source CT, while 3D reconstruction was performed with Vitrea Extend 6.7 graphic analysis system. The prominent features including: (1) Two branches of the medial basal segmental artery, AX^7^a and AX^7^t, emanated from the anterior segmental artery (A^8^) and the posterior segmental artery (A^10^), respectively (**Figure 2A**). (2) The medial basal subsegmental bronchi, classified as BX^7^a+BX^7^t according to Zhang M et al., originated from both the anterior (B^8^) and posterior (B^10^) segmental bronchi (**Figure 2B**). (3) The medial basal segmental vein (V^7^) shared a trunk with the anterior basal segmental vein (V^8^) and the lateral basal subsegmental vein (V^9^a) (**Figure 2C**).

**Figure 3:**
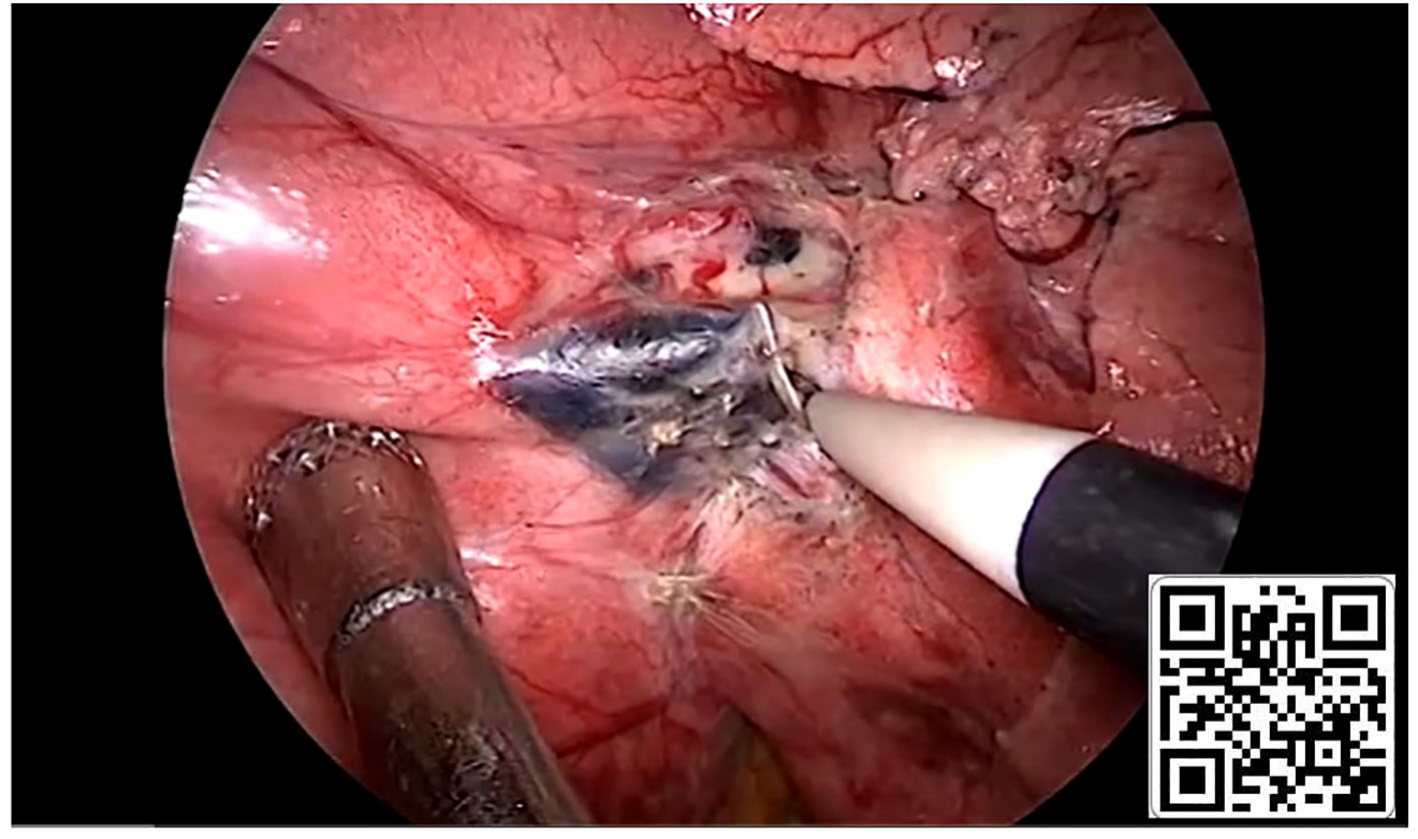
QR code for the surgical video. The surgical process can be obtained via scanning the QR code.

The tumor was diagnosed as stage cIA2 (cT1bN0M0). Anatomical pulmonary segmentectomy was the preferred protocol according to the National Comprehensive Cancer Network (NCCN) lung cancer guidelines (V2.2024). Based on detailed preoperative planning, thoracoscopic RS^8^ segmentectomy was performed precisely. Initially, the A^8^ and AX^7^a were clearly exposed after opening the interlobar fissure; the A^8^ was dissected using silk, while the AX^7^a was preserved (**Figure 2A**). Subsequently, after removing the lymph nodes surrounding the bronchi, the B^8^ was dissected using a stapler, and the BX^7^a was protected (**Figure 2B**). Thereafter, the V^8^ locating posterior to the B^8^ was exposed and ligated with silk (**Figure 2C**). Finally, the RS^8^ was divided using endostaplers, with the RS^7^a being successfully retained. The tumor was located 2.5 cm away from the incisal margin (**Figure 1B**). Intraoperative pathology analysis identified invasive adenocarcinoma, with intrapulmonary lymph nodes and surgical margins negative. Postoperative pathology analysis confirmed invasive adenocarcinoma with no involvement of the surrounding lymph nodes. The immunohistochemistry revealed that the Ki-67 index was 15%, which suggests slower tumor growth and more favorable prognosis. Additionally, the TTF-1 expression was strong positivity (+++), which supports the diagnosis of adenocarcinoma and indicates a higher likelihood of responsiveness to targeted therapies, should they be required in the future. The surgical process can be obtained via scanning the QR code (**Figure 3**). Following discharge, the patient underwent regular follow-up evaluations at six-month intervals. As of the most recent assessment in March 2025, there was no evidence of tumor recurrence or complications related to the surgery.

## Discussion

Recently, anatomic pulmonary segmentectomy has been widely performed for early-stage non-small cell lung cancer, in accordance to the National Comprehensive Cancer Network (NCCN) lung cancer guidelines (4). Understanding the anatomical structure of the pulmonary segments is essential for surgery, while 3D-CTBA technology has been proved to be highly beneficial in facilitating anatomic pulmonary segmentectomy. As reported by Chen H et al. (5), 3D-CTBA guided surgery demonstrates a reduction in both operation time and surgery-related complications compared to 2D-CT. In addition to that, Zhang M et al. (3) analyzed the economic impact of 3D-CTBA in thoracic surgeries, concluding that its ability to reduce surgical errors offsets the initial costs associated with imaging technology. Segmentectomy of the S^8^ tends to be considered more simple than other types of basal segmentectomies (6). However, it can also present a major challenge for surgeon when complex anatomical variations involved. This case reported a rare and complex anatomical variation in the basal segment of the RLL. To the best of our knowledge, such variation has rarely been reported. The success of this operation lies in resecting the RS^8^ precisely while preserving the RS^7^a.

The RS^7^ has the smallest volume of all pulmonary segments, but the anatomical structure of RS^7^ has been reported more complicated. Nagashima T et al. (7) classified the branching pattern of the B^7^ into four types:(B^7^a), (B^7^b), (B^7^ab) and (BX^7^). In this classification, the B^7^ is designated as BX^7^ when it does not originate from the base of the basal bronchus. Similarly, the branching pattern of the A^7^ has been divided into four types:(A^7^a), (A^7^b), (A^7^ab) and (AX^7^). Shimizu K et al. (8) reported that the incidence of such combined variant was 5.6%(15out of 270 cases). Zhang M et al. (3) firstly proposed an alternative classification for B^7^, identifying six distinct types: B^7^a, B^7^p, B^7^o, B^7^t, BX^7^a, and BX^7^t. When BX^7^ originates from B^8^, it is designated as BX^7^a, while when it derives from B^10^, it is referred to as BX^7^t. According to this classification, our case was categorized as the type of BX^7^a and BX^7^t. However, the variant we reported was more complex than previously documented. Two branches, designated as BX^7^a and BX^7^t, concurrently exist and originate from B^8^ and B^10^, respectively. The BX^7^a traversed anterior to the inferior pulmonary vein (IPV) and shared a trunk with B^8^, whereas BX^7^t passed through the IPV and originated from B^10^. Intraoperatively, (B^8^+ BX^7^a) can be easily mistaken for B^8^ because of such unique anatomical variation.

The anatomical variation of pulmonary vessels often coexists with bronchial variation, thereby complicating segmentectomy procedures, as reported by Gossot D et al. (9). This aligns with the current case, where such variations required precise surgical techniques. In this particular case, the A^7^ was composed of AX^7^a and AX^7^t, originating from A^8^ and A^10^, while V^8^ shared a trunk with V^7^ and V^9^a. Intraoperatively, such variations can be confusing for surgeons, potentially leading to the inadvertent transection of AX^7^a and V^9^a. The unique anatomical variation reported in this case, specifically the coexistence of BX^7^a and BX^7^t originating from B^8^ and B^10^ respectively, increases surgical difficulty due to the complexity of identifying and preserving critical structures like AX^7^a and V^9^a during segmentectomy. Misidentification of BX^7^a as part of B^8^ intraoperatively can lead to inadvertent transection of vital vessels or bronchi, emphasizing the need for meticulous preoperative 3D-CTBA planning and intraoperative judgment. In conclusion, this case presented a unique variation of bronchus and pulmonary vessels in the basal segment of the RUL. With the guidance of detailed preoperative planning, thoracoscopic RS8 segmentectomy was performed accurately.

## Data Availability

The original contributions presented in the study are included in the article/Supplementary Material. Further inquiries can be directed to the corresponding authors.
